# Supplementary material for: A high-resolution mRNA expression time course of embryonic development in zebrafish
Source: eLife. 2017 Nov 16;6:e30860. doi: 10.7554/eLife.30860 (PMC5690287; doi:10.7554/eLife.30860)
Supplement: Supplementary file 6. [file elife-30860-supp6.zip › biolayout-clusters-files/Cluster100-genes.html]

Cluster100


# Cluster100: Genes

| Ensembl ID | Gene Name | Chr | Start | End | Biotype |
| --- | --- | --- | --- | --- | --- |
| ENSDARG00000074528 | PRTFDC1 (1 of many) | 24 | 6627983 | 6641761 | protein\_coding |
| ENSDARG00000075161 | defbl1 | 21 | 21705168 | 21706355 | protein\_coding |
| ENSDARG00000035309 | entpd3 | 16 | 8344474 | 8367156 | protein\_coding |
| ENSDARG00000012366 | fbp2 | 5 | 44213911 | 44229566 | protein\_coding |
| ENSDARG00000039963 | fgfbp1b | 14 | 47454536 | 47455378 | protein\_coding |
| ENSDARG00000038056 | fgfbp2b | 1 | 21995222 | 22000443 | protein\_coding |
| ENSDARG00000004358 | gnb3a | 16 | 12131641 | 12158860 | protein\_coding |
| ENSDARG00000077236 | hspb6 | 15 | 37657985 | 37806875 | protein\_coding |
| ENSDARG00000058556 | muc5.2 | 25 | 8330824 | 8368332 | protein\_coding |
| ENSDARG00000105039 | phka1b | 21 | 30567097 | 30611411 | protein\_coding |
| ENSDARG00000037598 | prf1.5 | 8 | 29691731 | 29698007 | protein\_coding |
| ENSDARG00000093237 | si:ch211-89o9.4 | 1 | 40751423 | 40761220 | protein\_coding |
| ENSDARG00000069495 | tmem179b | 21 | 25627788 | 25633215 | protein\_coding |
